# Arctic coastal benthos long-term responses to perturbations under climate warming

**DOI:** 10.1098/rsta.2019.0355

**Published:** 2020-08-31

**Authors:** Amalia Keck Al-Habahbeh, Susanne Kortsch, Bodil A. Bluhm, Frank Beuchel, Bjørn Gulliksen, Carl Ballantine, Domiziana Cristini, Raul Primicerio

**Affiliations:** 1 Faculty of Biosciences, Fisheries and Economics, UiT- The Arctic University of Norway, Hansine Hansens veg 18, 9019 Tromsø, Norway; 2 Research Department, The Norwegian Polar Institute, Fram Centre, Hjalmar Johansens gate 14, 9009 Tromsø, Norway; 3 Environmental and Marine Biology, Åbo Akademi University, Tykistökatu 6, FI-20520, Turku, Finland; 4 Akvaplan-Niva, Fram Centre, Hjalmar Johans gate 14, 9009 Tromsø, Norway; 5 Aquatic Ecology and Evolution, Limnological Institute, University of Konstanz, Universitätsstraße 10, 78464, Konstanz, Germany

**Keywords:** community ecology, climate change, recolonization, succession, functional traits

## Abstract

Climate warming influences structure and function of Arctic benthic ecosystems. Assessing the response of these systems to perturbations requires long-term studies addressing key ecological processes related to recolonization and succession of species. Based on unique time-series (1980–2017), this study addresses successional patterns of hard-bottom benthos in two fjords in NW Svalbard after a pulse perturbation in 1980 and during a period of rapid climate warming. Analysis of seafloor photographs revealed different return rates of taxa, and variability in species densities, through time. It took 13 and 24 years for the community compositions of cleared and control transects to converge in the two fjords. Nearly two decades after the study initiation, an increase in filamentous and foliose macroalgae was observed with a subsequent reorganization in the invertebrate community. Trait analyses showed a decrease in body size and longevity of taxa in response to the pulse perturbation and a shift towards small/medium size and intermediate longevity following the macroalgae takeover. The observed slow recovery rates and abrupt shifts in community structure document the vulnerability of Arctic coastal ecosystems to perturbations and continued effects of climate warming.

This article is part of the theme issue ‘The changing Arctic Ocean: consequences for biological communities, biogeochemical processes and ecosystem functioning’.

## 1. Introduction

Arctic marine ecosystems are experiencing unprecedented environmental change due to climate warming and expanding human activities [1,2]. Climate change is causing temperatures in the Arctic to rise at over twice the global average, resulting in rapidly retreating sea ice [1,3]. Sea ice loss influences Arctic marine life by altering underwater light regimes, habitat availability, hydrography and ocean circulation [4–6]. The impact of climate warming can be particularly severe in coastal waters, where the above pressures are compounded by increased freshwater discharge and associated runoff from rivers and thawing glaciers [2,7,8]. Further, reduced ice cover increases the accessibility of Arctic waters, promoting the expansion of human activities [9] and increasing the associated risks of anthropogenic disturbances to marine communities.

The few existing Arctic long-term studies indicate recent reorganization of benthos resulting from climate-driven changes [7,10–12]. Observed changes include shifts in benthic biomass [6,13,14] and community composition [7,15], affecting respiration [16] and benthic-pelagic coupling [17,18]. In coastal hard-bottom habitats, such reorganization of benthic communities has been characterized by a sudden increase in foliose macroalgae, linked to altered light regimes due to sea ice loss, followed by structural changes in macrozoobenthos [7]. Other Arctic coastal studies find an increase in kelp biomass and a shift in the vertical distribution of kelp to shallower depths, resulting from reduced disturbance from ice-scouring, an extended ice-free period and increased turbidity [11,12]. This upward shift in macroalgae distribution is paralleled by increases in biomass and production of macrozoobenthos in the upper sublittoral zone [13]. Given the broad distribution of macroalgae along hard-bottom coasts across the Arctic [19,20], increases in cover, biomass and associated biodiversity constitute a facet of the ‘new Arctic' ecosystem functioning [8].

Understanding the mechanisms underlying ecological succession, i.e. the change in a community following a perturbation, is a prerequisite for separating natural variability from human disturbance effects in dynamic coastal systems [21]. The processes involved can be investigated *in situ* by combining taxonomic and functional traits approaches [22]. Colonization rates of high-latitude systems are generally much slower than at lower latitudes [10,23]. Yet, colonization patterns observed in other regions appear to hold also in the Arctic, with comparatively faster growing species colonizing more readily than slower growing, and often longer lived, species with a full benthic life cycle [24,25]. Furthermore, early colonizers may either be motile taxa that can migrate to disturbed areas to graze on newly established recruits [10], or short-lived sessile opportunists [26]. The latter often have poor competitive abilities. Strong competitors, which are typically larger and longer lived, dominate in later successional stages [24].

Earlier polar studies document slow recovery rates after disturbance in coastal areas and abrupt community shifts related to climate warming, implying that benthos are vulnerable to environmental change and perturbations. The recent expansion of human activities in the Arctic stresses the urgent need to understand why benthos is susceptible to environmental pressure. Here, we address the mechanisms shaping the recolonization and succession patterns underlying benthic responses to environmental perturbation, by combining taxonomic and trait-based (i.e. functional) approaches applied to temporal replicate samples that span four decades (1980–2017). Specifically, we compare community structure of hard-bottom benthos in control and manipulated (i.e. cleared) transects in two Arctic fjords to assess the rate and character of the recolonization in response to a pulse perturbation, through substrate clearance, and a press perturbation from climate change. We expect slow (i.e. decadal long) recolonization and recovery rates due to the slow growth and life cycles of larger invertebrates that tend to dominate later successional stages. Further, we expect a marked structural change related to climate warming, due to the changing habitat characteristics and macroalgae takeover favouring different functional groups.

## 2. Methods

### (a) Study sites

**Figure 1.**
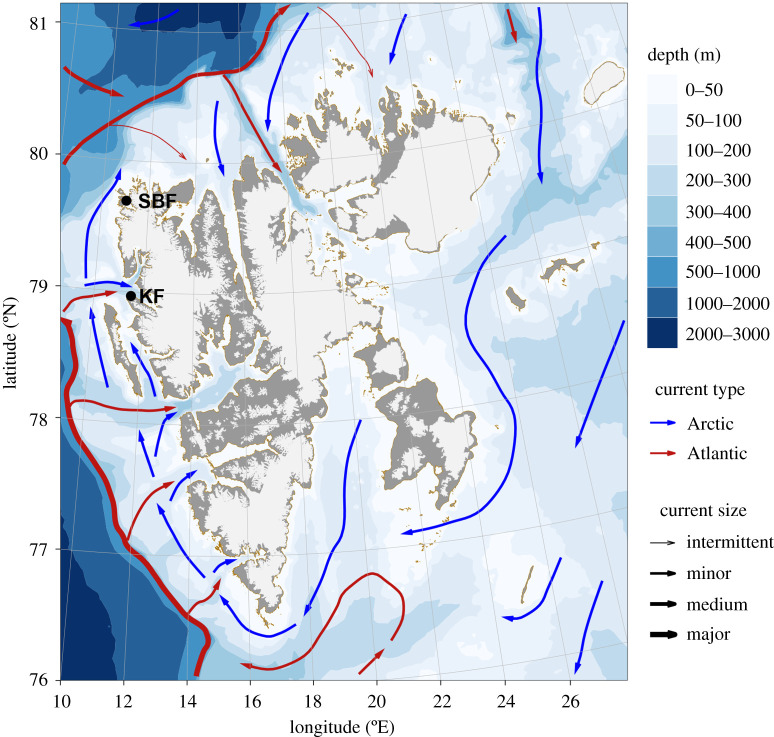
Location of study sites at Smeerenburgfjorden (SBF) and Kongsfjorden (KF) in the Svalbard Archipelago and general ocean current circulation. (Online version in colour.)

**Figure 2.**
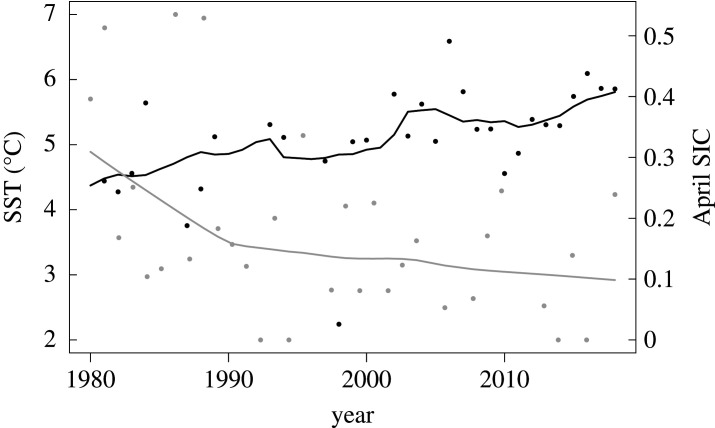
Decadal (black line) and annual (black points) average Sea Surface Temperature (SST,°C) from the West Spitsbergen Current, which influences the two study sites. Annual average April Sea Ice Concentration (SIC in percentage, grey points), and LOWESS smoother (grey line), close to the Smeerenburgfjorden site at 79°38'24′′ N, 11°18'36′′ E is estimated based on satellite imagery data made available from the Norwegian Polar Institute's Environmental monitoring of Svalbard and Jan Mayen (MOSJ). Temperature data were also obtained from MOSJ.

**Figure 3.**
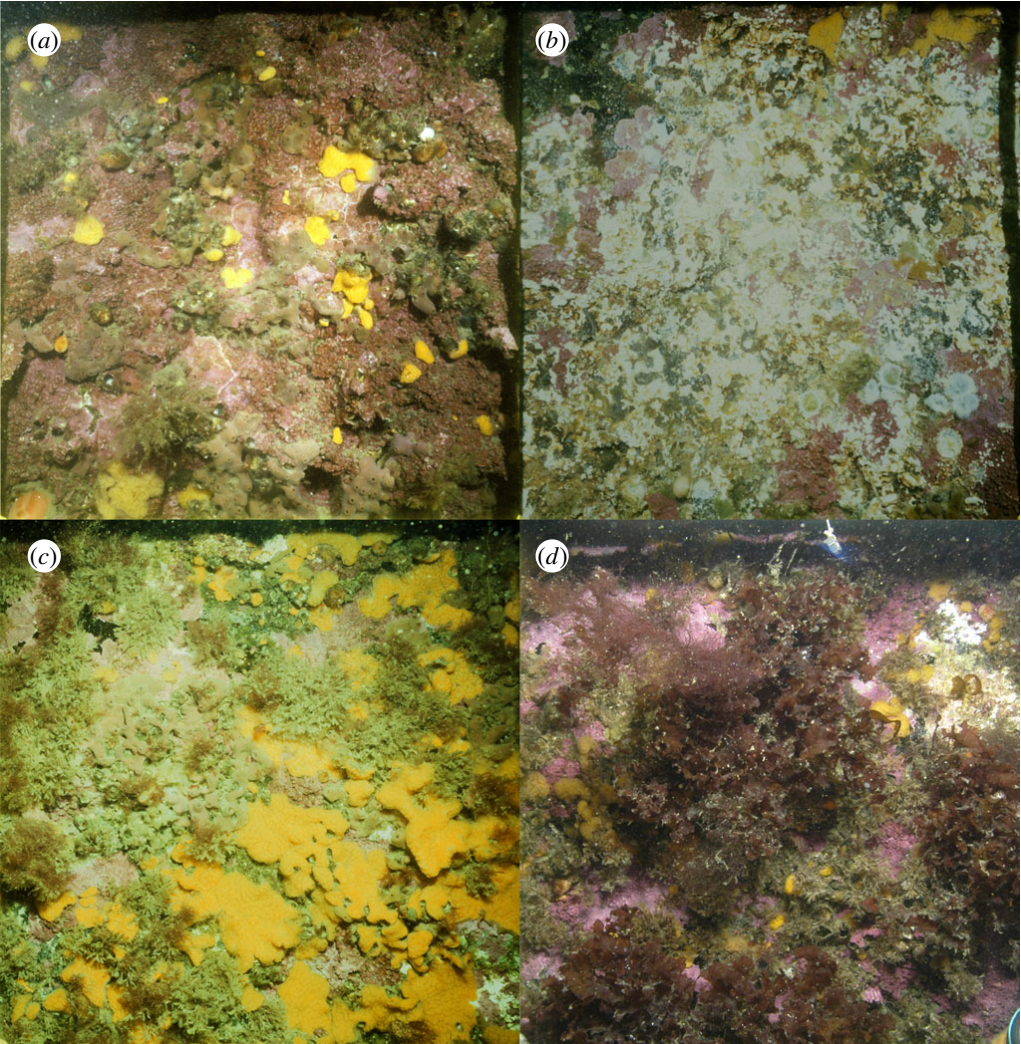
Hard-bottom community at study site Smeerenburgfjorden in the year 1980 before (*a*) and after (*b*) clearing of substrate. Recolonization of the same site in year 1994 (*c*) and 2008 (*d*), before and after macroalgae became dominant. (Online version in colour.)

The Smeerenburgfjorden and Kongsfjorden fjords are located on the northwest coast of the Svalbard Archipelago (figure 1), where the West Spitsbergen Current (WSC) advects warm saline water northwards as an extension of the North Atlantic Current [27]. This region has experienced increasing seawater temperature and reduced sea ice concentration over the study period (figure 2). Both sampling sites (Smeerenburgfjorden 79°41.3′ N, 11°04.0′ E; Kongsfjorden, 78°58.6′ N, 11°30.1′ E) are located near the coast at 15 m depth on hard-bottom habitats, in the outer parts of the fjords. The two sites differ by (i) their angle of inclination, with the Smeerenburgfjorden site being located on a vertical rock wall and the Kongsfjorden site on a horizontal bedrock bottom and by (ii) the influence of the WSC, with which the unsilled Kongsfjorden has a greater water exchange compared to the silled Smeerenburgfjorden. Both sites were established by SCUBA divers in 1980, each site consisting of two parallel transects of five adjacent 50 × 50 cm quadrats marked by bolts drilled into the bedrock, ensuring resampling of the same area in consecutive years. At each site, one transect was manipulated by clearing organisms and substrate (figure 3*a* before and *b* after manipulation), whereas the remaining transect was left untouched to serve as a control. Note that red crustose coralline algae could not be completely removed due to their strong adherence to the substrate (see electronic supplementary material, figure S1). The quadrats were then photographed annually by members of the author team and/or divers from UiT, The Arctic University of Norway, during cruises in August and September (figure 3*c* and *d*). A detailed description of the experimental and sampling design is provided in Beuchel *et al*. [28].

### (b) Image and taxonomic data analyses

Data acquisition is based on image analysis performed on seafloor photographs taken in Smeerenburgfjorden every other year between 1980 and 2017, and in Kongsfjorden every year between 1980 and 2004, thereafter every other year. Data from manipulated and control sites in Kongsfjorden for the years 1980–2003 are from Beuchel and Gulliksen [10], whereas the control transect data from Smeerenburgfjorden for the years 1980–2010 are from Kortsch *et al*. [7]. All remaining data were generated as part of the present study. To assess community structure, a semi-automated image analysis method in Adobe Photoshop, following Beuchel *et al*. [28], was applied to quantify the abundance of solitary taxa (individuals per m^−2^) and the percentage area covered by macroalgae and colony-forming taxa. Invertebrate and macroalgal taxa were identified from photographs based on a photographic reference collection combined with physical voucher material obtained from the study sites, in addition to the decade-long taxonomic expertise of the authors. Taxonomic names were standardized according to the World Register of Marine Species (WoRMS) (see electronic supplementary material, Tables S1 and S2 for full taxonomic lists).

For descriptive statistics and graphical summaries, data from the five adjacent images composing each transect were pooled. The time-series of individual taxa are presented only for taxa inhabiting Smeerenburgfjorden, for details on Kongsfjorden see [10,29]. Analyses of temporal change in benthic community structure for control and manipulated transects in each fjord were performed using correspondence analysis (CA), which relies on Chi-squared distances, well suited for count and percentage data [30]. Abundance and cover data were weighted differently in the CA to adjust for the uneven contribution to total inertia.

For inferential statistics, the quadrats captured by individual images were used as sampling units, providing five samples of benthic communities per transect. The effects of treatment, time and their interaction on community structure in each fjord were analysed by non-parametric multivariate ANOVA, applying ADONIS on Chi-square distance matrices [31]. Following the two-way ADONIS, pairwise comparisons of manipulated and control transects on each sampling year were performed via ADONIS and analysis of similarity ANOSIM [32], also based on Chi-squared distances, to investigate the recovery time of cleared communities, here defined as time to convergence of the manipulated community with the control community. The pairwise comparisons were performed on data for all taxa, and for a selection of taxa excluding rare, large, motile ones, which might affect dissimilarity between transects in ways that are unrelated to the process of community recovery after clearance (for details, see electronic supplementary material, tables S5–S8), as well as the small bristle worm, *Spirorbis spirorbis*, highly abundant in later years. Resemblance between transects was quantified with the ANOSIM R statistic, which varies between -1 and 1, with values close to 0 implying high similarity between communities. All non-parametric multivariate statistical tests were based on permutations. Shifts (discontinuities) in community structure were investigated by constrained classification of the control transect time-series within each fjord using sequential hierarchical clustering (CONISS algorithm) [33] on Chi-squared distances and multivariate regression trees (MRT) with time as the explanatory variable [34].

To address spatial mechanisms of recolonization, the position of individual members of the three taxa chitons (*Tonicella* spp.), the solitary ascidians, *Dendrodoa aggregata*, and barnacles (*Balanus balanus*), was registered in the manipulated transects in Smeerenburgfjorden. This allowed us to infer whether these taxa gradually recolonized the cleared areas from the edges or rapidly occupied the whole area.

### (c) Biological traits analyses

Information on biological traits was compiled from the online databases The Arctic Traits Database [35], BIOTIC [36], The Genus Traits Handbook [37], and from published literature, following guidelines by Degen and Faulwetter [35] and Bremner *et al*. [38,39] (see electronic supplementary material, table S9). The traits and trait modalities are consistent with the Arctic Traits Database, a repository of Arctic benthic invertebrate traits [35]. The chosen traits were body size, longevity, motility, living and feeding habit, sociability, dispersal, reproduction and development mode; only size and longevity are presented and discussed in detail here since these traits are closely related to colonization and succession and showed the largest changes over time. Size ranges are defined after The Arctic Traits Database [35] as small (less than 10 mm), small–medium (10–50 mm), medium (50–100 mm), medium–large (100–300 mm), large (greater than 300 mm) and indeterminate (clonal animals with unlimited growth). Whenever trait information for a given taxon was unavailable, information from taxa of a higher rank was gathered. Each trait modality was coded as 1 if expressed by a given taxon, and 0 if not expressed; species could display more than one modality for a given trait. This approach is similar to the commonly used fuzzy coding procedure [40]. To identify which traits were involved in the different stages of recolonization, a community-weighted trait analysis was applied by constructing an area-weighted traits-per-year matrix [39]. This approach allows us to track community level changes in trait composition and address ecosystem functioning under the assumption that dominant functional traits have the greatest effect on the ecosystem [41]. Only the invertebrate community from Smeerenburgfjorden was considered for the trait analysis.

All data analyses and figures were produced in R version 4.0.0 [42]; the geographical map was created with the ‘PlotSvalbard' R package [43]; CA were computed with the R package ‘ca’ [44]; ADONIS and ANOSIM relied on the R package ‘vegan' [45]. Sequential clustering (CONISS) and regression tree analyses (MRT) were computed using the R packages ‘rioja' [46] and ‘mvpart' [47], respectively.

## 3. Results

**Figure 4.**
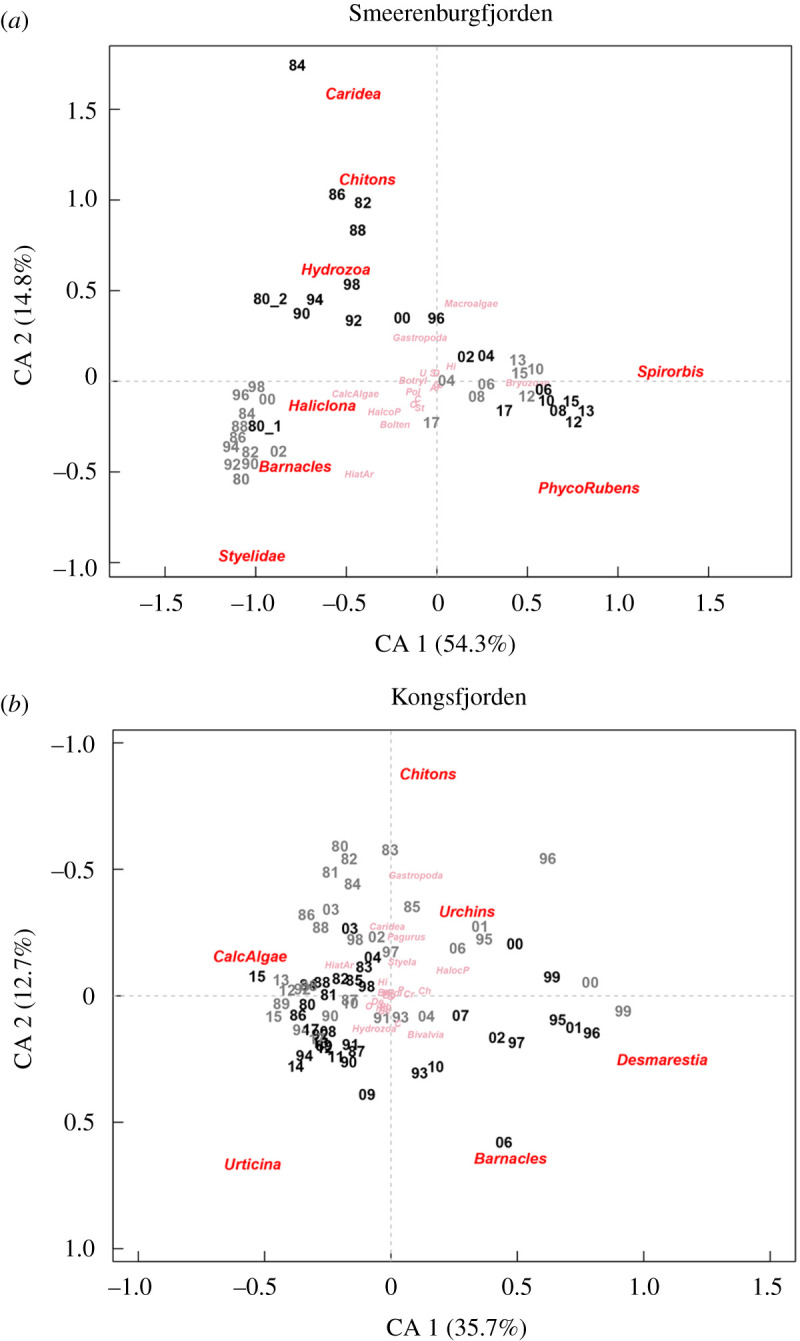
Correspondence analysis biplots of benthic community structure over the period 1980 to 2017 (numbers 80–17 correspond to years) in the cleared (black) and control (grey) areas in Smeerenburgfjorden (*a*) and Kongsfjorden (*b*). In both fjords, the first dimension is associated with climate-driven, temporal variation in community structure, and the second dimension with the effects of manipulation and recolonization. Taxa labelled in red are contributing most to the observed variation, whereas those in pink are of less importance. See electronic supplementary material, SI table 1 and 2 for abbreviations. (Online version in colour.)

**Figure 5.**
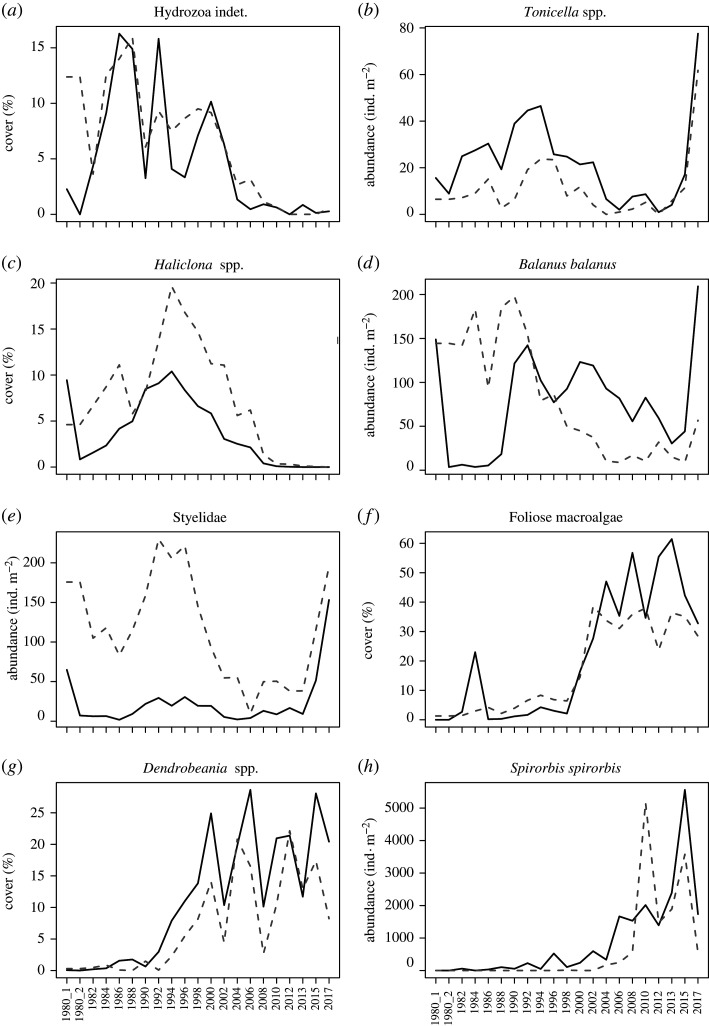
Temporal change in density of taxa from Smeerenburgfjorden in control (grey stippled line) and manipulated (black solid line) transects during the period 1980 to 2017. Shown are examples of different response patterns among taxa: (*a*,*b*) taxa with fast recovery rates after the pulse disturbance, (*c*–*e*) taxa with slower recovery rates, (*f*) abrupt increase in macroalgae and (*g*–*h*) increase in invertebrates associated with macroalgae takeover. In 1980, sampling took place before (1980_1) and after (1980_2) manipulation.

In both fjords, recovery after the pulse perturbation in 1980 took over a decade and was followed by a rapid reorganization associated with the climate-driven environmental change, as summarized by the CA results (figure 4). In Smeerenburgfjorden, the first two dimensions of the CA accounted for 69% of the total variation in benthic community structure among the control and manipulated transect (figure 4*a*). The first CA dimension, accounting for 54% of the variation, was associated with the climate-driven temporal changes in community structure, which displayed a marked and sudden shift after 2000, detected as a major discontinuity by sequential clustering and MRT (for detailed results, see electronic supplementary material, figures S2–S3). The second CA dimension, accounting for 15% of the variation, was related to differences between control and manipulated transects, with the cleared transect trajectory eventually converging towards the control. The effects on community structure were statistically significant for time (*p* < 0.01), treatment (*p* < 0.01) and their interaction (*p* less than 0.01), the latter indicating convergent trajectories of cleared and control communities (ADONIS table in electronic supplementary material, table S3). Pairwise comparisons confirmed a long recovery time (approx. 18 years) of the cleared community. This was indicated by the ANOSIM analysis (electronic supplementary material, table S6), where the lowest dissimilarity between transects (associated with a non-significant difference) was detected in 1998. In the CA biplot, manipulated and control community structure converged, in 2004, 24 years after the manipulation. This convergence coincided with the sudden increase of foliose macroalgae, primarily the red alga *Phycodrys rubens,* which was accompanied by rapid increments in invertebrates such as moss animals (*Dendrobeania* spp.) and the calcareous bristleworms (*S. spirorbis*) (figure 5*f–h*). The community of the cleared transect exhibited larger inter-annual variability (larger distance between years in the CA biplot) during the first two decades of recolonization and succession compared to the more stable control community (closely clustered years in the CA biplot). The two transects displayed similar patterns and trends from the year 2004 and onwards, illustrated by the strongly overlapping positions of the control and manipulated communities in the CA biplot. The patterns and trend in Kongsfjorden (figure 4*b*) were largely consistent with those in Smeerenburgfjorden, with significant effects of treatment (*p* < 0.01), time (*p* < 0.01) and their interaction (*p* < 0.01) on community structure (ADONIS table in electronic supplementary material, table S4). In Kongsfjorden, it took 13 years for the manipulated community to converge with the control community, with lowest dissimilarity and non-significant difference between transects registered in 1993 (electronic supplementary material, table S8), thereafter the benthic community underwent a sudden structural change detected as a main discontinuity in 1995 by both sequential clustering and MRT (electronic supplementary material, figures S4–S5). The first two dimensions of the CA accounted for 49% of the observed variation in community structure among control and cleared transects (figure 4*b*), with the first axis, capturing 36% of variation, being associated with the climate-driven change, and the second axis, accounting for 13% of the variation, being related to the differences between the control and cleared transects.

Taxa of the manipulated transect displayed different rates of recolonization. In Smeerenburgfjorden, early colonizers arrived 2 years after clearing of the substrate and included sessile hydrozoans and solitary motile chitons of the genus *Tonicella* spp. (figure 5*a,b*), and to a lesser degree, gastropods of the genus *Margarites* spp. and the shrimps Caridea (electronic supplementary material, figure S1). Densities of these taxa were high, but variable, during the first two decades and could exceed those of the control area. By contrast, sponges (porifera) of the genus *Haliclona* spp. (figure 5*c*) and barnacles (figure 5*d*) required more time (about a decade) to recolonize with densities comparable to those of the control community. Other taxa recolonized even slower; for example, abundance of the solitary ascidians *Halocynthia pyriformis* (electronic supplementary material, figure S1) and Styelidae (i.e. *Dendrodoa aggregata* and *Styela rustica*) (figure 5*e*) were much lower in the cleared transect compared to the control for more than 20 years. The images showed that barnacles, chitons, and *D. aggregata* gradually recolonized the cleared transect from the outer edges of the cleared area. In Kongsfjorden, patterns were generally comparable, but with some differences in the taxa appearing after the manipulation due to a different species composition at this site. Chitons and gastropods *Margarites* spp. were again the first to appear, but also sea urchins (*Strongylocentrotus* spp.) colonized quickly, whereas barnacles and sea anemones (*Urticina eques* and *Hormathia nodosa*) took longer to recover (figure 4*a*). For more details on taxon-specific dynamics and recovery rates after the pulse perturbation in Kongsfjorden, see [10].

In the years 1995 (Kongsfjorden) and 2000 (Smeerenburgfjorden), community structure in both cleared and control transects underwent drastic reorganizations (figures 4 and 5, electronic supplementary material, figure S2–S4). Most taxa had recovered from the manipulation by then. Taxa that had not previously been present, or present only in small numbers, suddenly occurred in high numbers or cover. In Smeerenburgfjorden, the strong increase in macroalgae cover was particularly conspicuous, primarily of the red alga *P. rubens*, followed by an increase in coverage of bryozoans (moss animals), and abundance of the calcareous tube-forming polychaete *S. spirorbis* (figure 5*f*–*h*). The community composition changed substantially, and a higher biodiversity was observed after the regime shift induced by the macroalgae [29]. In Kongsfjorden, the increase of the brown macroalgae *Desmarestia* spp. and sea urchins was accompanied by a rapid decline of sea anemones.

**Figure 6.**
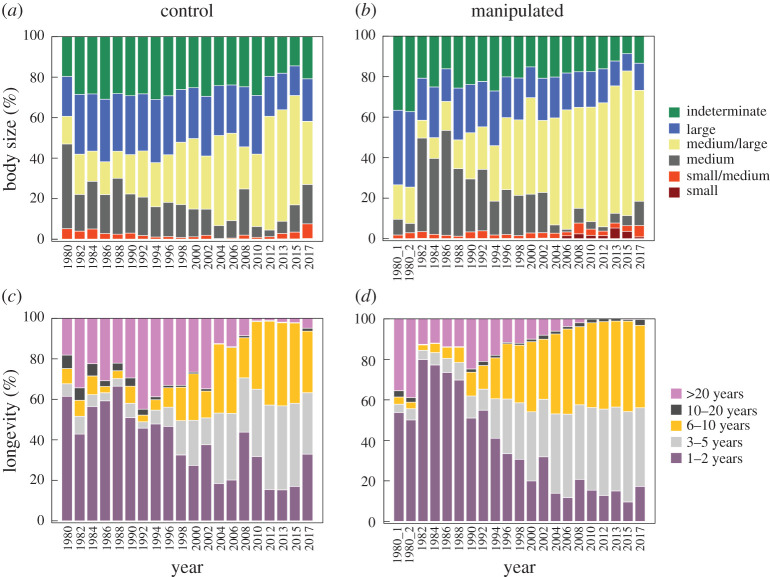
Temporal development of the body size (*a*,*b*) and longevity (*c*,*d*) composition in the benthic community of Smeerenburgfjorden in control (*a* and *c*) and manipulated (*b* and *d*) community from 1980 to 2017. The pulse perturbation was applied in 1980 and foliose macroalgae appeared in 2000. (Online version in colour.)

The recolonization of the cleared transects and the rapid reorganization in community structure following the sudden increase in macroalgae cover were associated with changes in trait modality composition of the benthic community (figure 6). In the years following the pulse perturbation and throughout the study, taxa with large body sizes and unlimited/indeterminate growth forms (i.e. sponges and colonial ascidians) were reduced in the cleared community compared to pre-disturbance and control communities (figure 6*a*,*b*). Following the press perturbation and sudden increase in foliose macroalgae, small/medium-sized taxa became more important in both cleared and control community, primarily due to the increase in bryozoans. Small-sized taxa increased in the cleared community due to the rising importance of spirorbid polychaetes. Following the pulse perturbation, short-lived taxa (1- to 2-year lifespans) increased in the cleared community, whereas long-lived taxa (10–20 and greater than 20-year lifespans) showed higher proportions in the control community (figure 6*c*,*d*). Beginning in the 1990s, and more markedly after 2000, taxa with intermediate (3–5 years and 6–10 years) lifespans increased in both cleared and control communities, with a subsequent reduction in long-lived taxa.

## 4. Discussion

Our long-term study of Arctic hard-bottom communities reveals a remarkable contrast between the slow recolonization rates following a pulse perturbation and the rapid reorganization associated with a climate-driven macroalgae expansion. The pioneers that colonized the pulse-perturbed area were medium-sized and short-lived taxa, such as hydrozoans and small chitons. These opportunistic taxa were slowly overtaken by longer lived barnacles, as well as larger and longer lived taxa like colonial sponges and ascidians. The abrupt increase in foliose and filamentous macroalgae in both manipulated and control areas of Smeerenburgfjorden was associated with reorganizations in invertebrate communities towards small/medium-sized and relatively fast-growing taxa, like moss animals and spirorbid polychaetes, whereas indeterminate growth form and long-lived taxa declined even further (e.g. sponges and ascidians). By providing food, substrate and shelter, foliose macroalgae likely facilitated colonization of associated taxa, explaining the fast reorganization of the benthic community. The climate-driven transition, mediated by the macroalgae takeover, towards communities with increased importance of faster growing, smaller sized taxa with higher turnover rates, will likely have implications for the dynamics and functioning of these Arctic ecosystems.

### (a) Recolonization

In line with our expectations, recovery rates of the two investigated Arctic benthic communities after substrate clearance were very slow, taking approximately 13 and 24 years in Kongsfjorden and Smeerenburgfjorden, respectively, before the community structure of cleared areas converged with those of control areas. The documented recovery rates in both fjords are substantially slower compared to lower latitudes but similar to those observed in other high-latitude systems. At lower latitudes, recovery times of benthic communities after substrate clearance are markedly shorter, amounting to 3 years in an intertidal hard-bottom habitat in South Africa [48] and 15 months in a soft-bottom habitat in sub-tropical Hongkong [49]. By contrast, recovery time after substrate clearance was projected to take over 10 years on boulders in the Beaufort Sea [50], and 10–14 years in soft-bottom communities from the Canadian Arctic Archipelago following an ice-scouring event [51]. An even longer recovery time was documented at Jan Mayen island (between Norway and Greenland), where a volcanic eruption in 1970 created new submarine lava grounds that took 30 years before it resembled ambient community structure [52]. In Antarctica, benthic recolonization after ice-scouring displays even slower recovery rates [53,54], with some estimates spanning hundreds of years [54]. In combination, these findings suggest that slow recolonization and recovery after disturbance is a general tendency for polar benthic ecosystems.

The longer recovery time of the Smeerenburgfjorden community compared to the Kongsfjorden community in this study also shows that, although slow, recovery rates can vary within a region, depending on local environmental conditions, the strength of biological and physical interactions, and local adaptations to stress [55]. The longer recovery time in Smeerenburgfjorden could, for example, be explained by its greater complexity in terms of number of species and ecological interactions [56]. By contrast, fewer species combined with higher sedimentation rates due to the horizontal surface orientation in Kongsfjorden characterize a community coping with a stronger regime of disturbance, leading to comparatively faster recovery rates. A similar pattern was indeed found at the Jan Mayen lava grounds study, where shorter recovery times (less than 8 years) were observed at shallower sites (5 and 10 m depth), compared to the deeper sites (15 m depth) [52]. The variation in recovery times was linked to differences in stress (from strong wave action, water currents and ice-scouring) and biodiversity.

The differences in recovery rates across latitudes may be linked to differences in temperature regimes between these regions (polar regions being colder), and its effect on species metabolism and life-history traits. Metabolic rates decrease as temperature decreases [57], and species with lower rates generally live longer, grow larger and more slowly and reproduce later in life compared to species with higher rates [58]. These differences in metabolic rates and life histories may explain why recovery rates in a colder, polar climate are much slower compared to faster recovery rates in a warmer climate at lower latitudes. These metabolic constraints may also explain why over the course of our study, occurrence of taxa with large (greater than 300 mm) body size and long lifespans (greater than 20 years) decreased, as temperature increased in the Arctic.

Although recovery rates vary between polar regions and lower latitudes, general successional patterns seem to be consistent across biogeographical areas. These patterns are attributable to differences in biological traits among benthic taxa. For example, early colonizers (chitons and hydrozoans) in this study are small and short-lived, traits that promote fast recolonization, confirming our *a priori* expectations. Colonial hydrozoans have previously been described as early colonizers following perturbations in Arctic habitats [24,59–62]. Other early colonizers in both the studied fjords, the motile grazing gastropods and chitons, may feed on small recruits of their co-colonizers such as hydrozoans, algae and bryozoans [10]. This is consistent with many temperate mollusc grazers preferring early successional macroalgae over later ones [63]. Motile taxa also appeared early after disturbance in Antarctic soft-bottom habitats, where amphipods and isopod crustaceans were observed to arrive within days after iceberg disturbance [64]. By contrast, taxa with slow colonization rates, including sponges and ascidians, are characterized as strong competitors yet late succession groups [24], consistent with their limited dispersal capacity, comparatively slower growth and higher longevity (figure 6). Communities of Antarctic sponges, for example, may need decades to fully recover from impact [65]. Also, their recolonization is often by vegetative encroachment [66], a slow process that in our study unfolded as a gradual expansion from the margins towards the centre of the cleared areas. The local competitive abilities of these sessile organisms are also related to their traits [67], reflected in large and longer lived taxa eventually overtaking small, short-lived opportunistic species. In conclusion, the very traits that boost competitive ability are often associated with limited capacity for rapid expansion and colonization [67]. While we document the spread of trait modalities within our fjord system, at the global level these polar invertebrates are generally characterized by slow growth and high longevity [10,38,39]. Given that ecosystems dominated by longer lived, k-strategist species often recover more slowly from perturbations than systems with r-strategists [68], these systems are particularly vulnerable to environmental and anthropogenic perturbations.

### (b) Ecological response to climate-driven increase in macroalgae

The fast reorganization of invertebrate communities following the sudden increase of macroalgae stands in stark contrast to the slow recolonization rates that followed the pulse perturbation in 1980. The rise in foliose macroalgae, registered in the years 1995 and 2000 in Kongsfjorden and Smeerenburgfjorden, respectively, has been linked to climate-driven reduction in sea ice cover and altered underwater light regimes [7,69]. Macroalgae facilitate benthic organisms by providing food, structural support and shelter, but can also inhibit other taxa by altering the light regime [11,13]. In a recolonization study from Greenland where canopy-forming algae were removed to study succession, associated fauna was facilitated by the macroalgae increasing habitat complexity and surface available for settlement [70]. In our study, similar facilitation effects may explain the appearance of filter-feeding bristle worms and moss animals in Smeerenburgfjorden. We suspect food availability could have been enhanced for the epibiotic spirorbids by being exposed to stronger water movement [71] in the uplifted position on the algal blades. The increase in moss animals and their ability to enhance macroalgae growth through nutrients excretion [72,73] could provide a feedback loop promoting further macroalgae expansion, a hypothesis that requires experimental testing in the future. Macroalgae can likely also impact other benthic organisms through negative effects such as shading and ameliorating wave actions and currents. This type of effect was documented in Kongsfjorden, where the increase in filamentous macroalgae led to sharp declines of sea anemones, which the authors explained by reduced water movement leading to increased sedimentation that clogged the feeding apparatus of the anemones [10]. We suspect that a combination of selective facilitation and inhibition of macroinvertebrates by the macroalgae helps explain the quick reorganization of benthic communities observed in the studied fjords.

### (c) Future Arctic coastal communities

The ongoing climate-driven change towards an increased importance of foliose and filamentous macroalgae in Arctic coastal waters raises questions on the future dynamics and functioning of these ecosystems. Macroalgae have the potential to greatly affect Arctic coastal hard-bottom systems, as primary producers, ecosystem engineers, and by engaging in ecological interactions with a broad range of species [20,70]. Macroalgae will likely provide an increasing food source in future Arctic coastal ecosystems and in detrital form already serve as a subsidy for food webs down to surprisingly great water depths [17,74]. Heterogeneity in energy pathways (i.e. phytoplankton, ice algae and macroalgae pathways) has been suggested to promote stability in food webs [75], including Arctic food webs [17,76]. It has thus been proposed that macroalgal detritus may dampen the effects of the strong seasonality in phytoplankton production, potentially leading to higher resilience of overall community production [17]. However, the relative importance of different food sources and energy pathways for the stability and functioning of Arctic food webs, and how these are coupled in time and space to higher trophic levels, remains elusive (but see [17,76]) and warrants further attention.

Increasing loss of sea ice cover and retreating glacial fronts continue to open new areas of hard-bottom habitats for colonization, especially in Svalbard, the Canadian Arctic Archipelago and Greenland [8]. Given that these rocky coasts constitute about a third of the Arctic coastline, which in turn contributes to approximately one third of the world's coastlines [77], the changing coastal communities will have large-scale effects on the associated ecosystems. Long-term time-series are an essential asset to study the response of these coastal communities to the escalating environmental change, given the slow growth and long lifespan of many Arctic benthic species. The low resilience and susceptibility to abrupt structural change of Arctic benthic communities raises concern over the ecological impact of future environmental change.

## Supplementary Material

Supplementary information

## Supplementary Material

Data files

## Data accessibility

Data is available as electronic supplementary material.
