# Supplementary material for: Arctic coastal benthos long-term responses to perturbations under climate warming
Source: Philos Trans A Math Phys Eng Sci. 2020 Aug 31;378(2181):20190355. doi: 10.1098/rsta.2019.0355 (PMC7481664; doi:10.1098/rsta.2019.0355)
Supplement: Supplementary information [file rsta20190355supp1.docx]

Supplementary material to “Arctic coastal benthos long-term responses to perturbations under climate warming”

Amalia Keck Al-Habahbeh, Susanne Kortsch, Bodil A. Bluhm, Frank Beuchel, Bjørn Gulliksen, Carl Ballantine, Domiziana Cristini, Raul Primicerio

Temporal development of abundance and cover of benthic taxa at Smeerenburgfjorden.


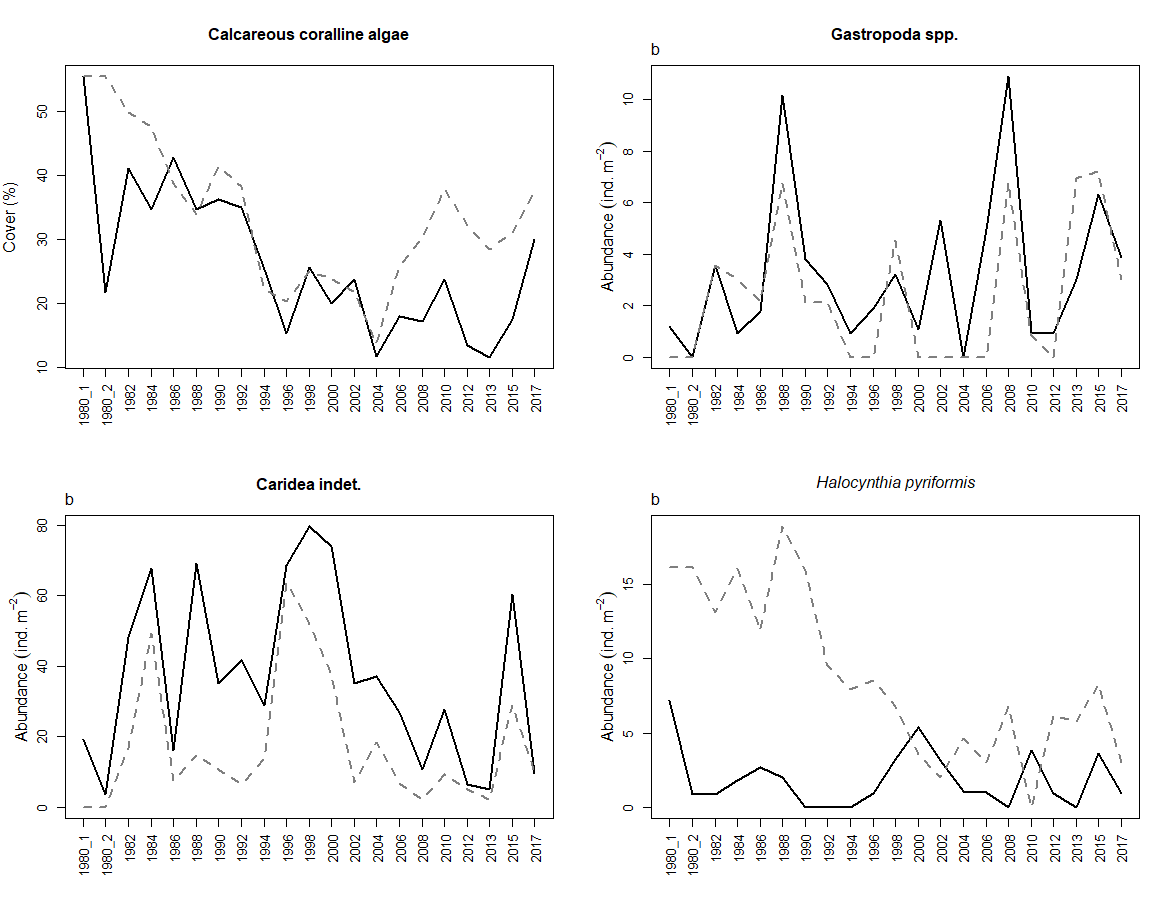


**Figure S1.** Additional univariate time series plots of taxa abundances and cover over time at Smeerenburgfjorden for calcareous coralline algae cover (%), Gastropoda indet., *Halocynthia pyriformis* and Caridea indet. (ind. per m^2^) in cleared (black solid line) and control (grey stippled line) areas in Smeerenburgfjorden.

Taxonomic lists

**Table S1.** Table with taxonomic level and Latin names, name abbreviations in the CA biplot, common names, and lifestyle (colonial/solitary) of invertebrates and macroalgae identified at study site in Smeerenburgfjorden.

|  | **Taxonomic level** | | **Identified taxa** | | | | **Abbreviation in biplot** | | **Common name** | | | **Colonial/solitary** | | | | | |
| --- | --- | --- | --- | --- | --- | --- | --- | --- | --- | --- | --- | --- | --- | --- | --- | --- | --- |
|  | Rhodophyta | |  | | | |  | |  | | |  | | | | | |
|  |  | Genus | *Lithothamnium* spp. | | | | CalcAlgae | | Crustose coralline algae | | | Colonial | | | | | |
|  |  | Genus | *Hildenbrandia* sp. | | | | Hi | | Crustose coralline algae | | | Colonial | | | | | |
|  |  | Class | Rhodophyta indet. | | | | Macroalgae | | Red algae | | | Colonial | | | | | |
|  |  | Species | *Euthora cristata* | | | | Macroalgae | |  | | | Colonial | | | | | |
|  |  | Species | *Savoiea arctica* | | | | Macroalgae | |  | | | Colonial | | | | | |
|  |  | Species | *Phycodrys rubens* | | | | PhycoRubens | |  | | | Colonial | | | | | |
|  |  | Species | *Turnerella pennyi* | | | | Macroalgae | |  | | | Colonial | | | | | |
|  |  | Genus | *Ptilota* spp. | | | | Macroalgae | |  | | | Colonial | | | | | |
|  | Ochrophyta | |  | | | |  | |  | | |  | | | | | |
|  |  | Class | Phaeophyceae indet. | | | | Macroalgae | | Brown algae | | | Colonial | | | | | |
|  |  | Species | *Saccorhiza dermatodea* | | | | Macroalgae | |  | | | Colonial | | | | | |
|  |  | Species | *Desmarestia aculeata* | | | | Macroalgae | | Mermaid’s hair | | | Colonial | | | | | |
|  |  | Species | *Desmarestia viridis* | | | | Macroalgae | | Sea sorrel | | | Colonial | | | | | |
|  | Porifera | |  | | | |  | |  | | |  | | | | | |
|  |  | Phylum | Porifera indet. | | | | P | | Sponge | | | Colonial | | | | | |
|  |  | Genus | *Haliclona* spp. | | | | Haliclona | |  | | | Colonial | | | | | |
|  |  | Genus | *Grantia* spp. | | | | G | |  | | | Colonial | | | | | |
|  | Cnidaria | |  | | | |  | |  | | |  | | | | | |
|  |  | Class | Hydrozoa indet. | | | | Hydrozoa | |  | | | Colonial | | | | | |
|  |  | Order | Actiniaria indet. | | | | A | | Sea anemone | | | Solitary | | | | | |
|  | Annelida | |  | | | |  | |  | | |  | | | | | |
|  |  | Species | *Spirorbis spirorbis* | | | | Spirorbis | |  | | | Solitary | | | | | |
|  |  | Class | Polychaeta indet. | | | | Pol | |  | | | Solitary | | | | | |
|  |  | Family | Polynoidae indet. | | | | Pol | | Scale worm | | | Solitary | | | | | |
|  |  | Class | Polychaeta indet., calcareous | | | | Pol | |  | | | Solitary | | | | | |
|  |  | Family | Sabellidae indet. | | | | Pol | | Feather duster worms | | | Solitary | | | | | |
|  | | Arthropoda | | | |  | | |  | | |  | |  | | |  |
|  | Crustacea | |  | | | |  | |  | | |  | | | | | |
|  |  | Species | *Balanus balanus* | | | | Barnacles | | Barnacle | | | Solitary | | | | | |
| Infraorder | | | Caridea indet. | | | | Caridea | | Shrimp | | | Solitary | | | | | |
|  |  | Genus | *Hyas araneus* and  *H. coarctatus* | | | | C | | Lyre crab and great spider crab | | | Solitary | | | | | |
|  |  | Infraorder | Brachyura indet. | | | | C | | Crab | | | Solitary | | | | | |
|  |  | Genus | *Pagurus* spp. | | | | C | | Hermit crab | | | Solitary | | | | | |
|  | Pycnogonida | |  | | | |  | |  |  | |  | | | | |  |
|  |  | Genus | *Nymphon* spp. | | | | S | | Sea spider | | | Solitary | | | | | |
|  | Mollusca | |  | | | |  | |  |  | |  | | | | |  |
|  |  | Species | *Hiatella arctica* | | | | HiaAr | | Wrinkled rock-borer | | | Solitary | | | | | |
|  |  | Genus | *Tonicella* spp. | | | | Chitons | | Chiton | | | Solitary | | | | | |
|  |  | Class | Gastropoda indet. | | | | Gastropoda | | Gastropods | | | Solitary | | | | | |
|  |  | Genus | *Margarites* spp. | | | | Gastropoda | |  | | | Solitary | | | | | |
|  |  | Species | *Chlamys islandica* | | | |  | | Iceland scallop | | | Solitary | | | | | |
|  | Bryozoa | |  |  | | |  | |  |  | |  | | | | |  |
|  |  | Genus | *Dendrobeania* spp. | | | | Bryozoan | |  |  | | Colonial | | | | |  |
|  | Echinodermata | | | | |  |  | |  | | |  | | | | | |
|  |  | Genus | *Henricia* spp. | | | | St | | Sea star | | | Solitary | | | | | |
|  |  | Species | *Ophiopholis aculeata* | | | | O | | Daisy brittle star | | | Solitary | | | | | |
|  |  | Species | *Ophiura* spp. | | | | O | | Brittle star | | | Solitary | | | | | |
|  |  | Genus | *Strongylocentrotus* spp. | | | | Urchin | | Green sea urchin | | | Solitary | | | | | |
|  |  | Genus | *Pteraster* spp*.* | | | | St | | Cushion star | | | Solitary | | | | | |
|  | Chordata | |  | | | |  | |  | | | | |  |  |  |  |
|  |  | Species | *Dendrodoa aggregata* | | | | Styelidae | |  | | | Solitary | | | | | |
|  |  | Genus | *Styela* *rustica* | | | | Styelidae | |  | | | Solitary | | | | | |
|  |  | Species | *Halocynthia pyriformis* | | | | HaloP | | Sea peach | | | Solitary | | | | | |
|  |  | Genus | *Botryllus* sp. | | | | Botryl | |  | | | Colonial | | | | | |
|  |  | Species | *Boltenia echinata* | | | | Bolten | | Cactus sea squirt | | | Solitary | | | | | |
|  |  | Species | *Didemnum albidum* | | | | D | | Northern white crust | | | Colonial | | | | | |
|  |  | Class | Ascidiacea indet. | | | |  | | Ascidian | | | Solitary | | | | | |

**Table S2.** Table with taxonomic level and latin names, name abbreviations in the CA biplot, common names, and lifestyle (colonial/solitary) of invertebrates and macroalgae identified at study site in Kongsfjorden.

|  | **Taxonomic level** | | **Identified taxa** | | | **Abbreviation in biplot** | | | **Common name** | | | **Colonial/solitary** | | | | | | | |
| --- | --- | --- | --- | --- | --- | --- | --- | --- | --- | --- | --- | --- | --- | --- | --- | --- | --- | --- | --- |
|  | Rhodophyta | |  | | |  | | |  | | |  | | | | | | | |
|  |  | Genus | *Lithothamnium* spp. | | | CalcAlgae | | | Crustose coralline algae | | | Colonial | | | | | | | |
|  |  | Genus | *Hildenbrandia* sp. | | | Hi | | | Crustose coralline algae | | | Colonial | | | | | | | |
|  |  | Species | *Phycodrys rubens* | | | Ph | | |  | | | Colonial | | | | | | | |
|  | Ochrophyta | |  | | |  | | |  | | |  | | | | | | | |
|  |  | Genus | *Desmarestia* spp. | | | Desmarestia | | | Brown algae | | | Colonial | | | | | | | |
|  | Porifera | |  | | |  | | |  | | |  | | | | | | | |
|  |  | Phylum | Porifera indet. | | | P | | | Sponge | | | Colonial | | | | | | | |
|  | Cnidaria | |  | | |  | | |  | | |  | | | | | | | |
|  |  | Class | Hydrozoa indet. | | | Hydrozoa | | |  | | | Colonial | | | | | | | |
|  |  | Order | *Urticina eques* | | | Urticina | | | Sea anemone | | | Solitary | | | | | | | |
|  |  | Species | *Gersemia rubiformis* | | | G | | | Sea strawberry | | | Colonial | | | | | | | |
|  | Annelida | |  | | |  | | |  | | |  | | | | | | | |
|  |  | Class | Polychaeta indet | | | Pol | | |  | | | Solitary | | | | | | | |
|  | | Arthropoda | | |  | | |  | | | |  | |  | | |  |  |  |
|  | Crustacea | |  | | |  | | |  | | |  | | | | | | | |
|  |  | Species | *Balanus balanus* | | | Barnacles | | | Barnacle | | | Solitary | | | | | | | |
| Infraorder | | | Caridea indet. | | | Caridea | | | Shrimp | | | Solitary | | | | | | | |
|  |  | Genus | *Hyas* spp. | | | C | | | Hy | | | Solitary | | | | | | | |
|  |  | Genus | *Pagurus* spp. | | | Pagurus | | | Hermit crab | | | Solitary | | | | | | | |
|  | Mollusca | |  | | |  | | |  |  | |  | | | | | |  | |
|  |  | Species | *Hiatella arctica* | | | HiaAr | | | Wrinkled rock-borer | | | Solitary | | | | | | | |
|  |  | Genus | *Tonicella* spp. | | | Chitons | | | Chiton | | | Solitary | | | | | | | |
|  |  | Class | Gastropoda indet. | | | Gastropoda | | | Gastropoda | | | Solitary | | | | | | | |
|  |  | Genus | *Margarites* spp. | | | Gastropoda | | |  | | | Solitary | | | | | | | |
|  |  | Species | *Chlamys islandica* | | | Ch | | | Iceland scallop | | | Solitary | | | | | | | |
|  |  | Class | Bivalvia indet. | | | Bivalvia | | | Iceland scallop | | | Solitary | | | | | | | |
|  |  | Genus | *Flabellina* sp. | | | F | | | Nudibranch | | |  | | | | | | | |
|  | Bryozoa  Phylum | | Bryozoa indet | | | Br | |  | Moss animal | | | Colonial | | | | |  | |  |
|  | Echinodermata | | | |  |  | | |  | | |  | | | | | | | |
|  |  | Genus | *Henricia* spp. | | | He | | | Sea star | | | Solitary | | | | | | | |
|  |  | Family | Ophiuridae indet. | | | O | | | Brittle star | | | Solitary | | | | | | | |
|  |  | Genus | *Strongylocentrotus* spp. | | | Urchins | | | Sea urchin | | | Solitary | | | | | | | |
|  |  | Genus | *Crossaster* sp. | | | Cr | | | Sun star | | |  | | | | | | | |
|  | Chordata | |  | | |  | | |  | | | | |  |  |  |  |  |  |
|  |  | Species | *Dendrodoa aggregata* | | | De | | |  | | | Solitary | | | | | | | |
|  |  | Genus | *Styela* spp. | | | Styela | | |  | | | Solitary | | | | | | | |
|  |  | Species | *Halocynthia pyriformis* | | | HaloP | | | Sea peach | | | Solitary | | | | | | | |
|  |  | Genus | *Botryllus* sp. | | | Bo | | |  | | | Colonial | | | | | | | |
|  |  | Species | *Boltenia echinata* | | | Be | | | Cactus sea squirt | | | Solitary | | | | | | | |

Nonparametric, multivariate ANOVA (ADONIS) of temporal and treatment effects on benthos community structure

For each fjord, the two-ways ADONIS was based on the chi-squared distance matrix, including all taxa in the analysis, with sequential test of terms by permutation (n=999).

**Table S3**. Nonparametric ANOVA (ADONIS) table for Smeerenburgfjorden

*Df SS MS F R2 P*

Treatment 1 77.301 77.301 38.988 0.1061 0.001

Year 19 195.840 10.307 5.199 0.2689 0.001

Treatment x Year 19 118.330 6.228 3.141 0.1624 0.001

Residuals 170 337.060 1.983 0.4626

Total 209 728.520 1.0000

**Table S4**. Nonparametric ANOVA (ADONIS) table for Kongsfjorden

*Df SS MS F R2 P*

Treatment 1 9.56 9.562 13.656 0.0278 0.001

Year 31 119.65 3.859 5.512 0.3479 0.001

Treatment x Year 31 35.41 1.142 1.631 0.1029 0.001

Residuals 256 179.26 0.700 0.5212

Total 319 343.88 1.0000

# **Resemblance analysis and pairwise tests of differences (ANOSIM and ADONIS) between manipulation and control transects**

**Tables S5-7.** Results of the resemblance analyses and pairwise tests of differences (ANOSIM and ADONIS) in the community structure between manipulation and control transects for each year. For each fjord, the permutation tests of ANOSIM and ADONIS were based on chi-squared distances; analyses were performed on all taxa, and on a selection of taxa excluding *Spirorbis spirorbis*, due to the high abundance of this species, as well as rare, motile taxa (crabs, brittlestars, brittle worms, starfish, seaspiders, and nudibranches)

**Table S5**. Pairwise comparisons (ANOSIM and ADONIS) of manipulated and control transects in Smeerenburgfjorden (all taxa included)

*Year R p_ANOSIM p_ADONIS*

1980 0.336 0.005 0.010

1980 0.400 0.006 0.011

1982 0.616 0.012 0.008

1984 1.000 0.012 0.006

1986 0.424 0.008 0.012

1988 0.812 0.007 0.009

1990 0.480 0.011 0.016

1992 0.468 0.014 0.006

1994 0.300 0.043 0.033

1996 0.536 0.011 0.026

1998 0.436 0.007 0.007

2000 0.416 0.026 0.022

2002 0.768 0.005 0.009

2004 0.552 0.015 0.017

2006 0.528 0.005 0.007

2008 0.528 0.011 0.008

2010 0.460 0.009 0.010

2012 0.728 0.017 0.014

2013 0.500 0.008 0.006

2015 0.536 0.013 0.011

2017 0.352 0.042 0.021

**Table S6**. Pairwise comparisons (ANOSIM and ADONIS) of manipulated and control transects in Smeerenburgfjorden (rare, motile taxa excluded)

*Year R p_ANOSIM p_ADONIS*

1980 0.384 0.004 0.011

1980 0.496 0.011 0.007

1982 1.000 0.007 0.005

1984 0.912 0.010 0.006

1986 0.768 0.011 0.011

1988 0.812 0.004 0.006

1990 0.332 0.025 0.028

1992 0.612 0.006 0.020

1994 0.384 0.021 0.034

1996 0.420 0.036 0.049

1998 0.216 0.072 0.028

2000 0.444 0.038 0.041

2002 0.600 0.008 0.007

2004 0.608 0.011 0.010

2006 0.612 0.007 0.012

2008 0.592 0.006 0.009

2010 0.608 0.010 0.004

2012 0.476 0.010 0.006

2013 0.708 0.008 0.011

2015 0.464 0.017 0.016

2017 0.664 0.010 0.012

**Table S7**: Pairwise comparisons (ANOSIM and ADONIS) of manipulated and control transects in Kongsfjorden (all taxa included)

*Year R p_ANOSIM p_ADONIS*

1980 0.532 0.009 0.007

1981 0.144 0.091 0.021

1982 0.368 0.020 0.014

1983 0.192 0.082 0.013

1984 0.636 0.012 0.008

1985 0.456 0.012 0.010

1986 0.316 0.017 0.012

1987 0.012 0.484 0.160

1988 0.272 0.039 0.033

1989 0.168 0.082 0.033

1990 0.132 0.095 0.052

1991 0.288 0.013 0.009

1992 0.364 0.012 0.015

1993 0.000 0.485 0.410

1994 0.184 0.089 0.047

1995 0.152 0.010 0.069

1996 0.376 0.024 0.013

1997 0.236 0.041 0.015

1998 0.136 0.124 0.135

1999 -0.028 0.617 0.594

2000 0.024 0.400 0.173

2001 0.192 0.051 0.107

2002 0.324 0.013 0.009

2003 0.136 0.106 0.044

2004 0.352 0.040 0.022

2006 0.440 0.014 0.009

2008 0.180 0.088 0.098

2010 0.228 0.016 0.050

2012 0.340 0.008 0.056

2013 0.524 0.006 0.009

2015 0.324 0.010 0.010

2017 0.268 0.012 0.024

**Table S8**. Pairwise comparisons (ANOSIM and ADONIS) of manipulated and control transects in Kongsfjorden (rare, motile taxa excluded)

*Year R p_ANOSIM p_ADONIS*

1980 0.496 0.004 0.012

1981 0.156 0.072 0.026

1982 0.368 0.014 0.022

1983 0.192 0.077 0.020

1984 0.624 0.006 0.009

1985 0.528 0.009 0.007

1986 0.360 0.017 0.008

1987 0.004 0.511 0.175

1988 0.288 0.052 0.037

1989 0.168 0.084 0.019

1990 0.152 0.091 0.045

1991 0.380 0.009 0.011

1992 0.332 0.030 0.043

1993 0.064 0.313 0.338

1994 0.104 0.161 0.123

1995 0.152 0.013 0.055

1996 0.348 0.037 0.026

1997 0.288 0.041 0.019

1998 0.136 0.154 0.142

1999 -0.004 0.520 0.571

2000 0.024 0.387 0.162

2001 0.160 0.068 0.107

2002 0.408 0.015 0.018

2003 0.108 0.151 0.087

2004 0.360 0.035 0.022

2006 0.428 0.016 0.010

2008 0.176 0.103 0.104

2010 0.292 0.017 0.047

2012 0.364 0.008 0.045

2013 0.560 0.007 0.014

2015 0.336 0.010 0.011

2017 0.168 0.044 0.042

# **Table S9**. Taxonomic names, as well as trait coding for each taxon in control and cleared transects: longevity, size, mobility, sociability, living habit, feeding mode, larval dispersal, reproduction mode, and development mode with reference numbers in parentheses. The references are provided below the table.

| **Taxa** | **Longevity** | **Size** | **Mobility** | **Sociability** | **Living habitat** | **Feeding mode** | **Larval dispersal** | **Reproduction mode** | **Development mode** |
| --- | --- | --- | --- | --- | --- | --- | --- | --- | --- |
| *Balanus balanus* | 6-10 & 10-20yr (1) | Small/medium (2) | No mobility (2) | Gregarious (2) | Attached (2) | Filter/suspension feeder (2) | >10km (3) | Sexual (2) | Planktotrophic (2) |
| *Boltenia* *echinata* | 3-5yr | Small/medium (2) | No mobility (2) | Solitary (2) | Attached No mobility (2) | Filter/suspension feeder (2) | 1-10m (est. from other solitary ascidians) | Sexual (2) | Lecithotrophic (4) |
| *Botryllus* spp. | 1-2yr (3) | Large, Indeterminate | No mobility | Colonial | Attached | Filter/suspension feeder | 1-10m (5) | Asexual (6) p. 10 | Lecithotrophic (3, 5) |
| *Dendrobeania* sp. | 3-10yr (7) | Small, small/medium (2) | No mobility (2) | Colonial (2) | Epiphytic/zoic, attached (2) | Filter/suspension feeder (2) | 1-100m (8) | Asexual & sexual (2) | Lecithotrophic (9) |
| *Dendrodoa aggregata* | 1-2yr (7) | Small/medium, medium (2) | No mobility (2) | Colonial (2) | Epiphytic/zoic, attached (2) | Filter/suspension feeder (2) | 1-100m (est. (7)) | Sexual & asexual (2) | Lecithotrophic |
| *Didemnum albidum* | 1-2yr (7) | Small/medium, medium (2) | No mobility (2) | Colonial (2) | Epiphytic/zoic, attached (2) | Filter/suspension feeder (2) | 1-10m (10) | Sexual & asexual (2) | Lecithotrophic (3) |
| *Grantia* spp. | >20yr (est. from *Haliclona* spp.) | Small/medium (2) | Attached | Colonial | Attached | Filter/suspension feeder | 1-100m (8) | Sexual (2) | Lecithotrophic (2) |
| *Halocynthia pyriformis* | 3-5yr (11) | Medium/large (2) | No mobility (2) | Solitary (2) | Attached (2) | Filter/suspension feeder (2) | 1-100m (est. from other ascidians) | Sexual (2) | Lecithotrophic (4) (2) |
| *Henricia* spp. | 3-5yr (3) | Medium/large, large (2) | Medium mobility (2) | Solitary (2) | Free-living (2) | Filter/suspension feeder, predator (2) | >10km (3) | Sexual (2) | Lecithotrophic (7) |
| *Hiatella arctica* | >20yr (12) | Small/medium (2) | Low (2) | Gregarious (2) | Burrowing, attached (2) | Filter/suspension feeder, predator (2) | >10km (7) | Sexual (2) | Planktotrophic (2) |
| *Hyas* spp. | 3-10yr (7) | Medium (2) | Low, medium (2) | Solitary | Free-living (2) | Surface/deposit, opportunist/scavenger, predator (2) | >10km (7) | Sexual (2) | Planktotrophic (13) |
| Hydrozoa indet. | 1-2yr (3) | Medium (2) | No mobility (2) | Colonial (2) | Attached (2) | Filter/suspension, predator (2) | 1-100m (3, 7) | Asexual (2) | Lecithotrophic (3) |
| *Margarites* spp. | 1-2yr (Arctic ocean diversity) | Small, small/medium (2) | Low mobility (2) | Solitary | Free-living, crevice-dwelling, epiphytic/zoic (2) | Surface/deposit, predator (2) | 1-10m (est. since brooder)  (14) | Sexual (2) | Lecithotrophic (15) |
| Caridea indet. | 3-10yr (1, 7) | Small/medium (2) | High (2) | Solitary | Free-living (2) | Predator (2) | >10km (7) | Sexual (2) | Planktotrophic (2) |
| Sea spider (*Nymphon* spp.) | 1-2yr (3) | Small, small/medium (2) | Medium (2) | Solitary | Free-living, epiphytic/zoic (2) | Predator (2) | 10-100m (3) | Sexual (2) | Planktotrophic (3) |
| *Ophiura* spp. | 3-10yr (7) | Small, small/medium (2) | Low, medium (2) | Solitary | Free-living. burrowing (2) | Surface/deposit, opportunist/scavenger, predator (2) | >10km (7) | Sexual (2) | Planktotrophic (7) |
| *Pagurus* spp. | 3-10yr (3, 7) | Small, small/medium (2) | Low, medium (2) | Solitary | Free-living, epiphytic/zoic (2) | Surface/deposit, filter/suspension, opportunist/scavenger, predator (2) | 1-10km (7) | Sexual (2) | Planktotrophic (9, 16) |
| Polychaeta indet | NA | Small/medium (2) | Low (2) | Solitary | Free-living (2) | Surface/deposit, filter/suspension, grazer, opportunist/scavenger, predator (2) | NA | Sexual, asexual (2) | Sexual, asexual (2) |
| *Pteraster* spp. | NA | Medium/large (2) | Medium/high | Solitary | Free living (2) | Predator (2) | NA | Sexual (2) | Lecithotrophic (8) |
| Actinaria indet. | >20yr (1) | Medium/large (2) | Low (2) | Solitary (2) | Attached (2) | Predator (2) | >10km (est. from 11-30 days larval duration (3) | Sexual & asexual (2) | Lecithotrophic (2) |
| *Spirorbis spirorbis* | 1-2yr (17) | Small (2) | No mobility (2) | Gregarious | Tube-dwelling, epiphytic/zoic, attached (2) | Filter/suspension feeder (2) | 1-100m (8) | Sexual (2) | Lecithotrophic (2) |
| *Haliclona* spp. | >20yr (18) | Large, indeterminate | No mobility (2) | Colonial | Attached (2) | Filter/suspension, predator (2) | 1-100m (8) | Sexual & asexual (2) | Lecithotrophic (19) |
| *Strongylocentrotus* spp. | >20yr (20) | Medium (2) | Low (2) | Gregarious (2) | Free-living (2) | Surface/deposit, grazer (2) | >10km (3) | Sexual (2) | Planktotrophic (3) |
| *Styela* spp. | 1-2yr (3) | Medium (2) | No mobility (2) | Solitary (2) | Attached (2) | Filter/suspension feeder (2) | 1-100m (3) | Sexual (2) | Lecithotrophic (3) |
| *Tonicella* spp. | 3-5yr (est. from *Lepidochitona cinereal*) (21) | Small/medium (2) | Low mobility | Solitary | Free-living | Surface/deposit, grazer (2) | 10-1000m (est from larval duration) (22) | Sexual (2) | Lecithotrophic (9) |

# Where no information on trait modalities was found in the literature for a given taxon, the trait was estimated from images of the present study and/or estimated from a closely related taxon.

# **References:**

1. Gulliksen B, Svensen E. Svalbard and life in polar oceans. Kristiansund: Kom; 2004.

2. Degen R, Faulwetter S. The Arctic Traits Database–a repository of Arctic benthic invertebrate traits. <https://www.univie.ac.at/arctictraits/> Earth System Science Data. 2019;11(1):301-22.

3. MarLIN, BIOTIC - Biological Traits Information Catalogue. <http://www.marlin.ac.uk/biotic/> Plymouth: Marine Biological Association of the United Kingdom. 2006.

4. Svane I, Lundälv T. Population dynamics and reproductive patterns of boltenia echinata (Ascidicea) on the Swedish west coast. Netherlands Journal of Sea Research. 1982;16:105-18.

5. Grosberg RK. Limited dispersal and proximity-dependent mating success in the colonial ascidian *Botryllus schlosseri*. Evolution. 1987;41(2):372-84.

6. Ali HAJ, Tamilselvi M. Ascidians in Coastal Water: A Comprehensive Inventory of Ascidian Fauna from the Indian Coast: Springer; 2016.

7. Handbook MMGT. Available from <http://www.genustraithandbook.org.uk/>

8. McEdward LR. Ecology of marine invertebrate larvae: CRC press; 1995.

9. Dethier MN, McDonald K, Strathmann RR. Colonization and connectivity of habitat patches for coastal marine species distant from source populations. Conservation Biology. 2003;17(4):1024-35.

10. Jackson J. Modes of dispersal of clonal benthic invertebrates: consequences for species' distributions and genetic structure of local populations. Bulletin of Marine Science. 1986;39(2):588-606.

11. Svavarsson J. Life cycle and population dynamics of the symbiotic copepod Lichomolgus canui Sars associated with the ascidian Halocynthia pyriformis (Rathke). Journal of experimental marine biology and ecology. 1990;142(1-2):1-12.

12. Sejr MK, Petersen JK, Jensen KT, Rysgaard S. Effects of food concentration on clearance rate and energy budget of the Arctic bivalve Hiatella arctica (L) at subzero temperature. Journal of Experimental Marine Biology and Ecology. 2004;311(1):171-83.

13. Anger K, Dawirs R. Influence of starvation on the larval development of Hyas araneus (Decapoda, Majidae). Helgoländer Meeresuntersuchungen. 1981;34(3):287.

14. Strathmann RR. Why life histories evolve differently in the sea. American Zoologist. 1990;30(1):197-207.

15. Hadfield MG, Strathmann MF. Heterostophic shells and pelagic devolopment in trochoideans: implications for classification, phylogeny and palaeoecology. Journal of Molluscan Studies. 1990;56(2):239-56.

16. Lovrich GA, Thatje S. Reproductive and larval biology of the sub-Antarctic hermit crab Pagurus comptus reared in the laboratory. Journal of the Marine Biological Association of the United Kingdom. 2006;86(4):743-9.

17. Ni S, Taubner I, Böhm F, Winde V, Böttcher ME. Effect of temperature rise and ocean acidification on growth of calcifying tubeworm shells (Spirorbis spirorbis): an in situ benthocosm approach. Biogeosciences. 2018;15(5):1425.

18. Teixidó N, Garrabou J, Harmelin J-G. Low dynamics, high longevity and persistence of sessile structural species dwelling on Mediterranean coralligenous outcrops. PloS one. 2011;6(8):e23744.

19. Carballo JL, Ávila E. Population dynamics of a mutualistic interaction between the sponge Haliclona caerulea and the red alga Jania adherens. Marine Ecology Progress Series. 2004;279:93-104.

20. Blicher ME, Rysgaard S, Sejr MK. Growth and production of sea urchin Strongylocentrotus droebachiensis in a high-Arctic fjord, and growth along a climatic gradient (64 to 77 N). Marine Ecology Progress Series. 2007;341:89-314.

21. Fish JD, Fish S. A Student's Guide to the Seashore. London: Unwin Hyman Limited; 1989.

22. Barnes J, Gonor J. The larval settling response of the lined chiton Tonicella lineata. Marine Biology. 1973;20(3):259-64.

Detection of temporal shifts (discontinuities) in benthos community structure by sequential clustering and Multivariate Regression Trees (MRT)


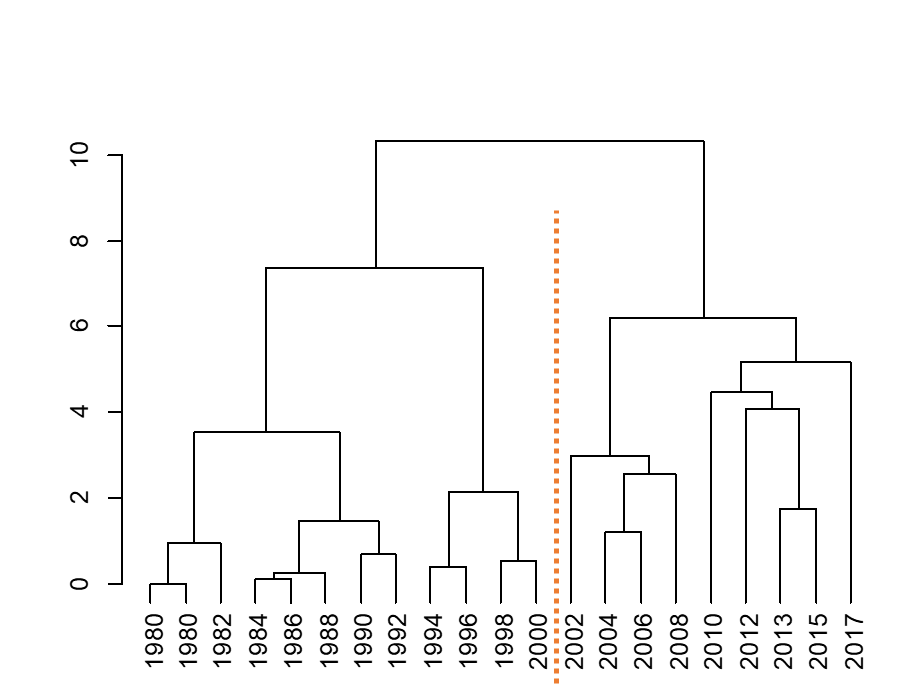


**Figure S2.** Sequential clustering dendrogram of benthic community in Smeerenburgfjorden. Red stippled line indicates a main discontinuity in benthos community structure associated with climate-driven macroalgae take-over after the year 2000.

**Figure S3.** Dendrogram of the Multivariate Regression Tree result for Smeerenburgfjorden displaying the timing of the main discontinuity in benthos community structure after the year 2000.


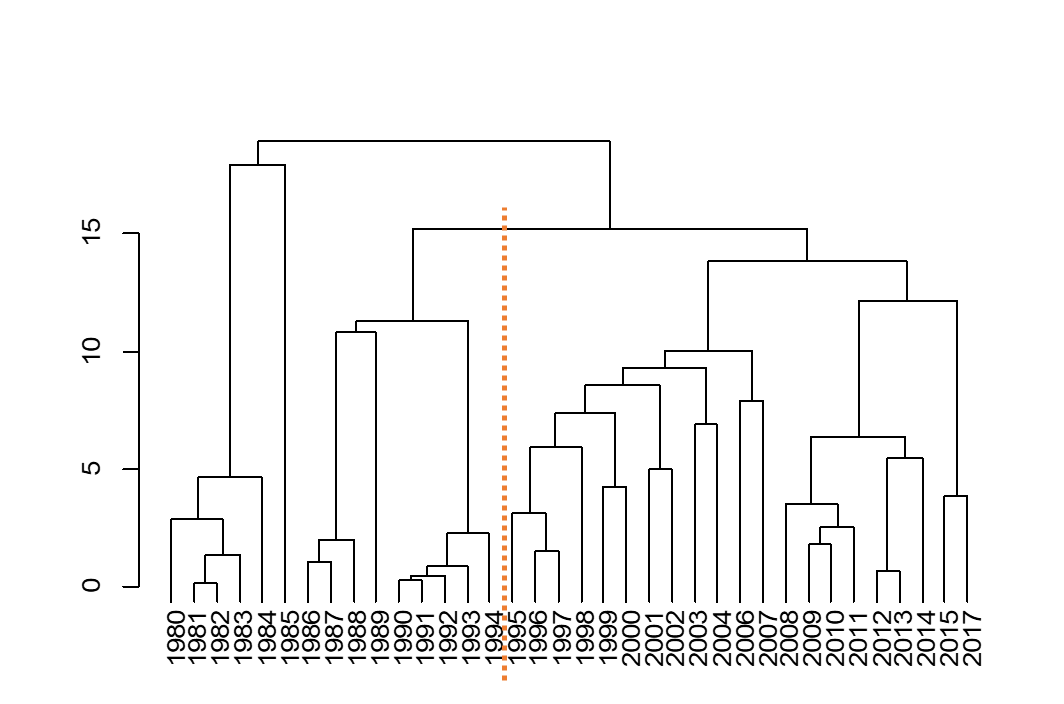


**Figure S4**. Sequential clustering dendrogram of benthic community in Kongsfjorden. Red stippled line indicates a main discontinuity in benthos community structure associated with climate-driven macroalgae take-over after 1994.

**Figure S5**. Dendrogram of the Multivariate Regression Tree result for Kongsfjorden displaying the timing of the main discontinuity in benthos community structure.
